# Industrial-grade nitrogen sources modulate CaCO_3_ polymorphs and strength in MICP-cemented sand: A structure–property study

**DOI:** 10.1371/journal.pone.0348780

**Published:** 2026-05-05

**Authors:** Yu Zhang, Xu Zhang, Bo Peng, Yongzhi Zhang, Zherui Chen, Guohui Ma, Jin Wang, Qiang Wen, Miao Liu

**Affiliations:** 1 China Three Gorges Corporation, Wuhan, China; 2 Three Gorges Jinsha River Yunchuan Hydropower Development Co., Ltd., Kunming, China; 3 School of Civil and Hydraulic Engineering, Huazhong University of Science and Technology, Wuhan, China; Soongsil University, KOREA, REPUBLIC OF

## Abstract

Microbially induced carbonate precipitation (MICP) is promising for soil stabilization. However, its large-scale application is hindered by the cost of laboratory-grade yeast extract (YE), which often accounts for more than 70% of cultivation medium expenses. Here, we evaluate two standardized industrial nitrogen sources—industrial yeast extract (IYE) and soy peptone (SP)—as complete or partial replacements for YE in *Sporosarcina pasteurii* cultivation. Urease activity and the performance of bio-cemented sand columns were assessed via unconfined compressive strength (UCS), CaCO_3_ content, and mineralogical/microstructural analyses. Results indicated that the partial substitution scheme of 5 g/L pure YE + 10 g/L IYE yielded the best overall outcomes: bacterial urease activity reached ~80% of the control, UCS reached 4.27 MPa (9% higher than the control), and the nitrogen-source cost was reduced by 65.53%. The enhanced strength correlates with a favorable precipitation pathway that produced predominant calcite (~95.57% of the CaCO_3_ precipitate), together with a dense, interlocking microstructure. In contrast, SP-substituted media produced lower UCS despite a high CaCO_3_ content (up to 15.31%), indicating that mechanical performance depends not simply on the total amount or final polymorph of CaCO_3_, but on the combined effects of polymorph composition, precipitation pathway, and the resulting microstructural organization. Overall, the proposed YE–IYE blending strategy offers a practical route to lower-cost, higher-performance MICP sand stabilization.

## 1. Introduction

Microbially induced carbonate precipitation (MICP) is an environmentally sustainable technique for stabilizing soils by harnessing microbial metabolism. In ureolytic systems, urease-producing bacteria (e.g., *Sporosarcina pasteurii*) catalyze urea hydrolysis, elevating alkalinity and supplying carbonate ions that react with calcium to precipitate calcite, thereby bridging and cementing loose grains [1]. Compared with conventional soil stabilization methods such as chemical grouting [2] and mechanical compaction [3], MICP exhibits distinct advantages, including mild reaction conditions (ambient temperature and pressure) and reduced energy consumption [4]. Moreover, the calcite generated through MICP is chemically analogous to natural minerals and exhibits high long-term environmental compatibility, making it attractive for applications such as slope stabilization, foundation reinforcement, and the restoration of historical buildings [5–8].

In parallel with the growing recognition of these advantages, MICP research has increasingly moved beyond laboratory demonstrations toward engineering-scale implementation. Cubic-meter outdoor trials and meter-scale radial grouting experiments have shown that scaled-up cultivation and injection strategies can achieve MPa-level strength while enabling control over CaCO_3_ distribution, thereby providing direct experimental support for field-scale application [1,9]. For practical deployment, research attention has also expanded to bacterial formulation and construction operability. In 2025, a dried, shelf-stable *S. pasteurii* formulation was reported to induce cementation within 24 h under field conditions and improve bearing capacity, highlighting the feasibility of standardized and product-oriented MICP applications [10]. Beyond terrestrial geotechnical applications, recent studies have begun to explore MICP for extraterrestrial construction, in which lunar or Martian regolith simulants are used as in situ raw materials to fabricate “space bio-bricks”, providing a potential strategy to reduce the prohibitive cost of transporting conventional construction materials from Earth [11].

As these application scenarios continue to broaden, the economic feasibility of MICP becomes an increasingly critical issue. Despite the extensive validation of MICP’s theoretical mechanisms and laboratory-scale efficacy, its large-scale engineering application remains constrained primarily by high operational costs. A substantial portion of these costs arises from bacterial screening and medium preparation. Nitrogen sources alone often account for over 70% of the total medium expenses [12–14]. Conventional nitrogen sources for nutrient media, such as high-purity yeast extract (YE) and nutrient broth, are prohibitively expensive, thereby undermining the economic feasibility of large-scale MICP implementation [15].

Consequently, research has shifted toward low-cost alternatives, primarily industrial and agricultural by-products such as corn steep liquor (CSL), whey, and sugarcane molasses [16,17]. These substrates have demonstrated strong potential to drastically reduce cultivation costs—sometimes by over 90%—while in some cases even enhancing urease activity or the compressive strength of bio-cemented samples [12,15,18–22]. However, this cost-saving advantage is fundamentally compromised by a critical flaw: inherent variability. These crude by-products possess complex, non-standardized compositions, which introduce uncontrolled organic components and contaminants [13,23–29]. This leads to inconsistent microbial metabolism, unpredictable CaCO_3_ precipitation kinetics, and ultimately, irreproducible biocementation outcomes—a major barrier to standardized, large-scale application.

Accordingly, cost reduction remains a central theme in recent MICP research. Authoritative reviews have summarized multiple economic routes, including the use of low-cost or food-grade nutrient sources and the substitution of urine or inexpensive calcium sources [30]. Subsequent work in 2026 further demonstrated, through statistical optimization, that the relationship among formulation, precipitation kinetics, and UCS can be systematically designed [31].

Meanwhile, the understanding of material performance is also shifting from quantity to quality. A 2025 review highlighted that medium composition, extracellular polymeric substances (EPS), and organic molecules can govern polymorph selection among calcite, vaterite, and other CaCO_3_ phases [32]. More recent studies further revealed that humic substances and yeast extract may simultaneously promote nucleation and complex dissolved ions, thereby altering crystallization pathways and indicating that engineering formulations must carefully balance additive dosage windows [33,34].

Therefore, to achieve reproducible and scalable MICP, there is a compelling need for alternative nitrogen sources that are both cost-effective and compositionally consistent. This study addresses this need by investigating two standardized, industrial-grade alternatives: soy peptone (SP) and industrial yeast extract (IYE). Their defined chemical profiles promise more predictable bacterial growth and metabolic activity compared to variable agro-wastes [35,36]. Crucially, beyond considerations of cost and consistency, a deeper scientific gap persists. While previous studies have evaluated such alternatives using broad metrics such as biomass yield and overall strength [12,21,29], their subsequent impact on the material-level properties of the bio-cement remains largely unexplored. The influence of these specific nitrogen sources on the micro-scale characteristics of the carbonate precipitate—such as its polymorph selection (calcite vs. vaterite), crystal morphology, and spatial distribution within the pore network—remains unclear. Furthermore, the mechanistic links between nitrogen source formulation, the resulting precipitate microstructure (e.g., crystal-pore interlocking), and macroscopic mechanical performance remain elusive. This lack of understanding impedes the targeted optimization of substitution strategies, particularly the balance between high-purity and industrial-grade sources in partial substitution regimes.

To address these gaps, this study aims to reduce MICP medium costs while enhancing material performance by systematically investigating the effects of replacing pure YE with industrial alternatives (SP and IYE) under both complete and partial substitution regimes. First, the urease activity of bacterial cultures was characterized using conductivity measurements. Subsequently, sand column biocementation experiments were conducted to evaluate how the optimized nutrient substitution schemes influence unconfined compressive strength (UCS), CaCO_3_ content, and the characteristics of the precipitated carbonate, including mineral phase composition and microstructural features such as crystal morphology and pore distribution in the treated sand. By integrating multi-scale characterization techniques, we elucidate the underlying mechanisms that link the nitrogen source formulation to the quality of the carbonate precipitates (in terms of phase and microstructure) and to the resulting mechanical performance. The ultimate objective is to identify nitrogen source substitution strategies that balance cost-effectiveness with high material performance, thereby providing theoretical and experimental support for the engineering application of the MICP technique.

## 2. Materials and methods

**Fig 1:**
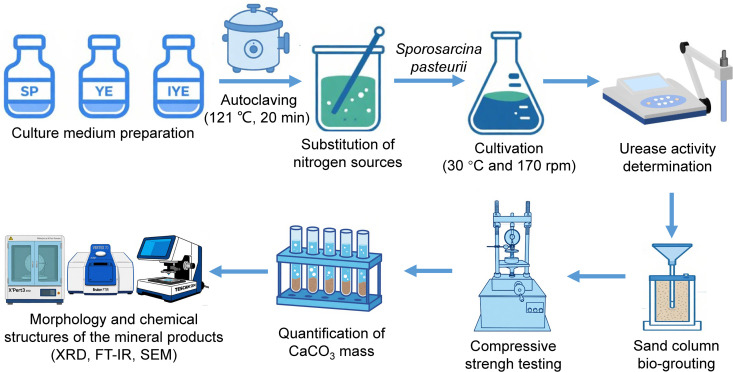
Experimental flowchart for low-cost nitrogen source substitution and multi-scale evaluation of sand columns. The process includes microbial cultivation with a urease activity assay, sand biocementation, and subsequent mechanical testing and microstructural characterization.

The experimental workflow was designed to evaluate the cost-effectiveness and biocementation performance of substituting pure yeast extract (YE) with low-cost alternatives: soy peptone (SP) and industrial-grade YE (IYE). As illustrated in Fig 1, the study consisted of two sequential phases: (1) microbial cultivation and urease activity assays under different nitrogen source substitution strategies, and (2) biocementation of sand columns and subsequent performance characterization.

### 2.1. Materials and nitrogen source characterization

The bacterial strain *Sporosarcina pasteurii* (BNCC 337394) was obtained from the BeNa Culture Collection (Suzhou, China). Nitrogen sources included analytical-grade pure YE (Oxoid, catalog no. LP0021B, 190 CNY/500 g), IYE (catalog no. Y017C, 36 CNY/500 g), and SP (catalog no. Y005C, 50 CNY/500 g). Both IYE and SP were purchased from Beijing Hongrun Baoshun Technology Co., Ltd. All other chemicals were of analytical reagent grade (≥ 99.0%), including (NH_4_)_2_SO_4_, NaOH, MnSO_4_·H_2_O, NiCl_2_·6H_2_O, HCl, urea, and CaCl_2_.

**Fig 2:**
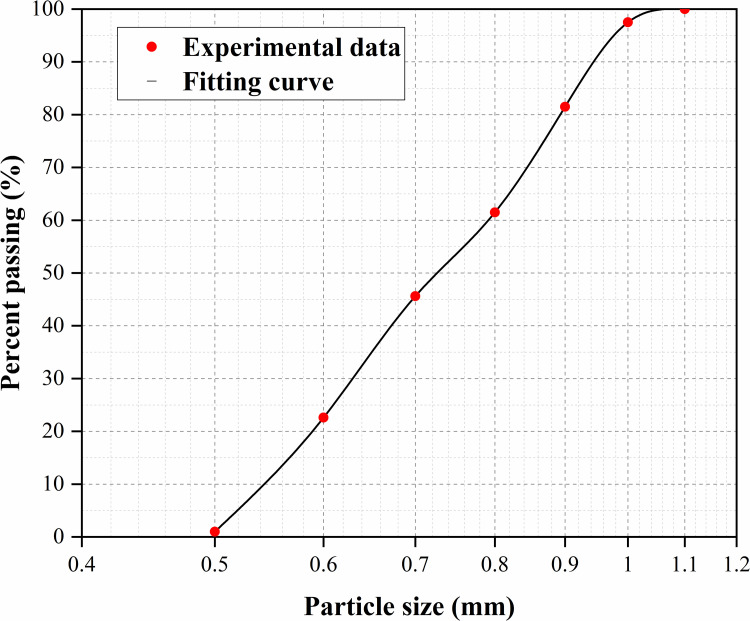
Particle size distribution of the test sand.

Quartz sand with a particle size of 0.5–1.0 mm was used as the aggregate for column construction. The particle size distribution is shown in Fig 2, and the basic physical properties are detailed in Table 1. Prior to media preparation, the solid powders of YE, IYE, and SP were characterized. Elemental composition (C, H, N, O, S) was quantified using a Vario MICRO cube elemental analyzer (Elementar, Germany). For each nitrogen source, three independent measurements were performed, and the values reported are the mean values (n = 3). The elemental analyzer provides stable and reproducible quantification for comparing the elemental composition of different nitrogen sources. UV–Vis spectra (200–600 nm) were acquired using a SolidSpec-3700 spectrophotometer (Shimadzu, Japan), while thermal stability was assessed via thermogravimetric analysis (TG/DTG) using an STA449F3 analyzer (NETZSCH, Germany), with heating from 40 to 950 °C under a nitrogen (N_2_) atmosphere.

**Table 1:** Basic physical properties of the test sand.

Specific Gravity Gs	D10 (mm)	D50 (mm)	Maximum Dry Density (g/cm^3^)	Minimum Dry Density (g/cm^3^)	Coefficient of Uniformity *C*_*u*_	Coefficient of Curvature *C*_*c*_
2.65	0.54	0.73	1.72	1.43	1.46	0.92

### 2.2. Nitrogen source substitution, microbial cultivation, and urease activity assay

Pure YE at 20 g/L was used as the control because this concentration is commonly adopted in *S. pasteurii* culture media, such as ATCC 1376 and DSMZ 220, and can reliably support bacterial growth and urease activity. The concentrations of the industrial nitrogen sources were not set by direct mass equivalence to YE, but were determined based on their compositional characteristics and preliminary screening tests. Because IYE is compositionally closer to YE, its complete substitution concentration was set at 20 g/L. In contrast, SP required a higher dosage to provide comparable growth support and was therefore set at 40 g/L for complete substitution. For partial substitution, 5 g/L YE was retained to support core metabolic activity, while 10 g/L IYE and 15 g/L SP were selected based on preliminary screening as representative levels that balance activity retention and cost reduction. Based on these criteria, five experimental groups were established for the biocementation tests (Table 2).

**Table 2:** Experimental groups for nitrogen source substitution.

Group	Nitrogen source	Concentration (g/L)	Substitution type	Relative cost (%)
**YE (Control)**	Pure YE	20	–	100
**SP**	SP	40	Complete	52.63
**YE + SP**	Pure YE + SP	5 (pure) + 15 (soy)	Partial	44.75
**IYE**	IYE	20	Complete	18.95
**YE + IYE**	Pure YE + IYE	5 (pure) + 10 (industrial)	Partial	34.47

The basal culture medium consisted of the designated nitrogen source, 10 g of (NH_4_)_2_SO_4_, and 1 mL/L of a trace element solution (12 g/L MnSO_4_·H_2_O and 24 g/L NiCl_2_·6H_2_O), with the pH adjusted to 8.5 using 1 M NaOH. The medium was sterilized by autoclaving at 121 °C for 20 min before use. Bacteria were incubated in the prepared medium at 30 °C and 170 rpm. Urease activity was measured at 16 h and 22 h post-inoculation using a conductivity-based method with a DDSJ-308F conductivity meter (Shanghai Yidian Scientific Instruments Co., Ltd.). The 16 h time point represents the active growth stage, whereas 22 h corresponds to the early stationary stage, at which urease activity was more representative of the bacterial suspension used for subsequent MICP treatment. These two time points were selected to compare nitrogen-source effects during both the growth-associated and high-activity stages. The bacterial suspension was mixed with 1.11 M urea solution (at a volume ratio of 1:9) at 30 °C, and the variation in electrical conductivity (ΔmS·cm^-1^·min^-1^) was recorded over 5 min. Urease activity (U) was calculated as [37]:


Activity (U) = ΔConductivity/min × 11.1 × 10 (1)

where 11.1 is the empirical conversion factor and 10 accounts for the tenfold dilution, U is expressed as mM urea hydrolyzed per minute (or its equivalent) based on the empirical conductivity-to-urea conversion.

### 2.3. Sand column preparation and MICP treatment protocol

Standard PVC molds with a diameter of 36.9 mm were filled with 130 g of sand (to a height of 80 mm) and compacted in two layers to form sand columns. The column assembly consisted, from bottom to top, of a rubber base, a porous stone, the sand column, and a perforated top plate. For each treatment group, four parallel sand columns were prepared. Three specimens were used for UCS testing, and the central portions of these specimens were subsequently used for CaCO_3_ content determination, while one additional specimen was reserved for microstructural characterization.

To investigate the effect of nitrogen source substitution on biocementation, five experimental groups were established (Table 2), following the substitution strategies outlined in Section 2.2. The cementation solution was prepared by mixing equal volumes of 1.0 M urea and 1.0 M CaCl_2_, yielding a final concentration of 0.5 M for each component.

A total of five MICP treatment cycles were conducted over 10 days. For each cycle, 30 mL of bacterial suspension, harvested after 22 h of cultivation and adjusted to OD600 = 1.2 after centrifugation and resuspension, was injected. This was followed by three injections of 30 mL of cementation solution at 12-h intervals, resulting in a total of 15 treatments with cementation solution. Equal volumes of 1.0 M urea and 1.0 M CaCl_2_ were mixed, yielding final concentrations of 0.5 M for both urea and CaCl_2_.

### 2.4. Performance evaluation of bio-cemented columns

#### 2.4.1. Physical and mechanical testing.

Prior to testing, both ends of the specimens were ground flat to ensure parallelism. The mechanical performance was then evaluated via unconfined compressive strength (UCS) tests using a strain-controlled triaxial apparatus (loading rate: 1 mm/min, loading accuracy: ± 0.5%) in accordance with the Standard for Geotechnical Test Methods (GB/T 50123−2019). For each group, three parallel specimens were used for UCS testing, while one additional specimen was reserved for microstructural characterization. UCS data are reported as mean ± SD (n = 3). Statistical differences among groups were analyzed using one-way ANOVA followed by Tukey’s HSD post hoc test. A p value < 0.05 was considered statistically significant. Following failure, the calcium carbonate (CaCO_3_) content was determined by acid washing. A representative sample (approximately 4 cm in height) was extracted from the column center and treated with 5% HCl. The content was calculated based on the dry mass difference before and after acid dissolution.

#### 2.4.2. Morphology and chemical structures.

X-ray diffraction (XRD) analysis was carried out using an X’Pert3 powder diffractometer (PANalytical B.V., Netherlands) to determine the crystal structure and phase composition of the bio-cemented sand. The instrument was equipped with a Cu Kα radiation source (λ = 1.540598 Å) and operated at 40 kV and 40 mA. Scans were performed from 20° to 60° (2θ) at a rate of 4°/min. Mineral phases were identified by matching diffraction patterns with reference standards from the ICDD PDF database. The relative calcite/vaterite fractions within the CaCO_3_ phase (excluding quartz) were estimated using the intensity-ratio calibration proposed by Kontoyannis and Vagenas [38]. Specifically, the peak intensities of calcite (104) at 2θ ≈ 29.4° and vaterite (110) at 2θ ≈ 27.0° were used. For binary calcite–vaterite mixtures, the intensity ratio follows the relationship:


$$\frac{I_{104}}{I_{110}}=7.691\times \frac{X_{C}}{X_{V}}$$ (2)

where I_110_ and I_104_ are the measured peak intensities, X_V_ and X_C_ are the molar fractions of vaterite and calcite, respectively (X_V_ + X_C_ = 1). Consequently, the molar fractions can be calculated.


$$X_{V}=\frac{7.691}{7.691+I_{104}/I_{110}}$$ (3)

$$X_{C}=1-X_{V}$$ (4)

The chemical composition of the precipitated minerals was analyzed with a Bruker Vertex 70 Fourier transform infrared (FT-IR) spectrometer (Germany). Measurements were performed in transmission mode using KBr pellets with 64 scans, a spectral resolution of 4 cm^-1^, and a wavenumber range of 4000–400 cm^-1^.

#### 2.4.3. Microstructure.

The morphology of the CaCO_3_ crystals was examined using a scanning electron microscope (SEM; TESCAN, Czech Republic) at an acceleration voltage of 5 kV and a working distance of 10 mm. Prior to SEM observation, samples were sputter-coated with a gold layer to prevent surface charging.

## 3. Results and discussion

### 3.1. Urease activity and cost analysis

**Fig 3:**
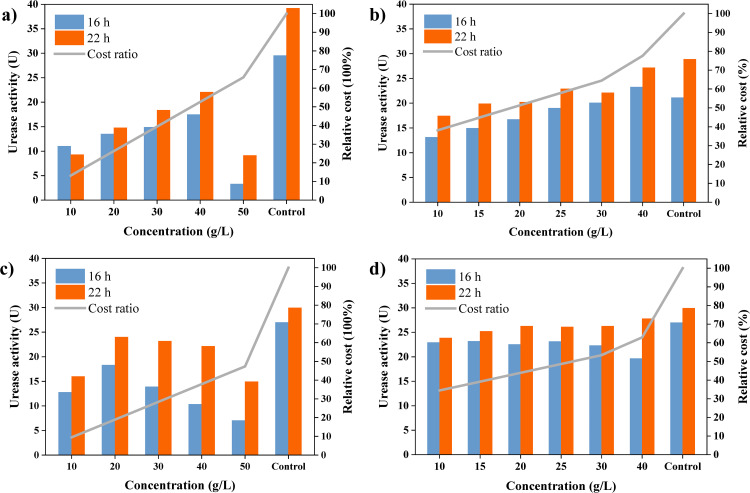
Urease activity and relative cost of nitrogen source replacement schemes. The control (YE) refers to the medium containing 20 g/L pure yeast extract (YE), for which the relative cost was defined as 100%. Panels (a–b) show SP-based replacement and panels (c–d) show IYE-based replacement under complete (a, c) and partial (5 g/L YE + substitute, b, d) substitution.

The influence of nitrogen source substitution on urease activity was quantified through electrical conductivity measurements (Fig 3). The results indicate that enzyme activity was strongly influenced by both the substitution strategy (complete versus partial) and the type of nitrogen source. Partial substitution, which incorporated a small quantity of pure YE to support core metabolic functions, was substantially more effective at maintaining urease activity than complete substitution. IYE exhibited particularly favorable performance under this strategy.

As shown in Figs 3(a) and 3(c), complete replacement of pure YE (control: 20 g/L, 27.75 U) led to a marked decline in urease activity. Among the substitutes evaluated, only SP at 40 g/L and IYE at 20 g/L exceeded 16.65 U. In contrast, partial substitution with 5 g/L pure YE supplemented with 10 g/L of the industrial nitrogen source significantly restored activity for both nitrogen sources, reaching levels comparable to the control (Figs 3(b) and 3(d)). Specifically, the combination of 5 g/L pure YE and 10 g/L IYE under partial substitution yielded optimal performance, achieving 80% of the control urease activity (22.2 U). These findings highlight the importance of including a small amount (5 g/L) of pure YE to sustain metabolic functionality and identify the partial substitution with IYE as the most cost-effective strategy for maintaining high urease activity.

In addition to enzymatic activity, an evaluation of the cost–performance trade-off underscores the practical benefits of these substitution strategies. The most substantial cost reduction was achieved by the complete substitution with IYE (IYE group), reducing the nitrogen-source cost to only 18.95% of the control. However, this came at the expense of significantly compromised urease activity. In comparison, the optimal partial substitution formulation (YE + IYE group) offered a favorable balance, achieving a 65.53% cost reduction while maintaining high urease activity—a critical factor for effective biocementation. This combination of considerable cost savings and sustained efficacy represents a key advancement for scalable MICP applications, facilitating the transition from laboratory research to economically feasible soil improvement technology.

### 3.2. Sand column strength

**Fig 4:**
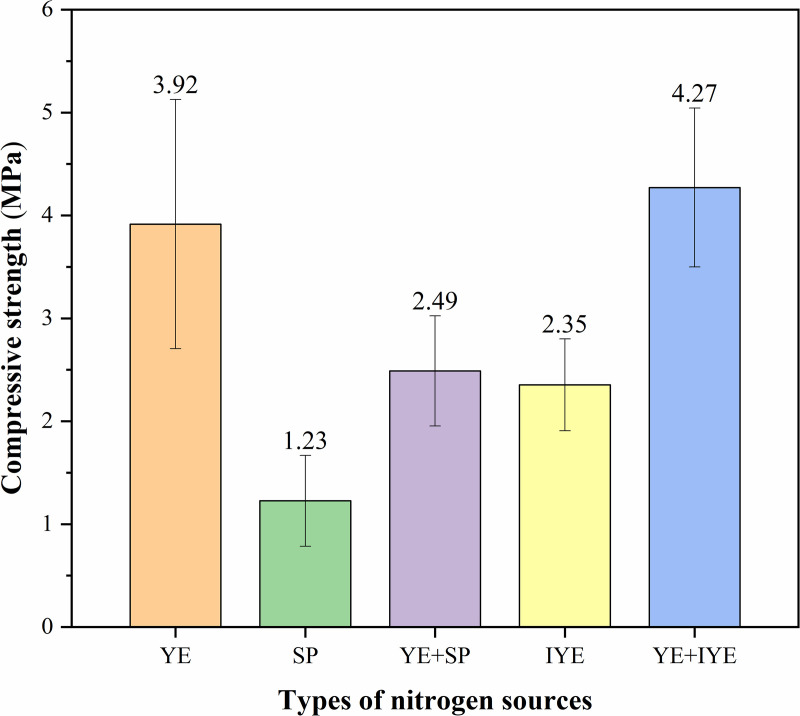
UCS of bio-cemented sand columns under different nitrogen source substitutions (mean ± SD, n = 3). (YE (Control): Pure YE (20 g/L); SP: SP (40 g/L, complete substitution); YE + SP: Pure YE (5 g/L) + SP (15 g/L, partial substitution); IYE: IYE (20 g/L, complete substitution); YE + IYE: Pure YE (5 g/L) + IYE (10 g/L, partial substitution)).

Overall, UCS generally increased with urease activity across the substitution strategies (Fig 4), although the relationship was not strictly linear among all groups. Except for the IYE group (2.35 MPa), all complete substitution groups exhibited low UCS values (< 2.0 MPa), which was broadly consistent with their relatively limited retained urease activities. Notably, the IYE-based partial substitution group (5 g/L pure YE + 10 g/L IYE) achieved the highest mean UCS (4.27 MPa), which was not significantly different from that of the control group (3.92 MPa) (p = 0.9762). The YE + SP group showed an intermediate UCS (2.49 MPa), consistent with its intermediate retained urease activity. Statistical analysis further indicated that only the SP group had a statistically lower UCS than the YE control (p = 0.0085), whereas the YE + SP and IYE groups were not significantly different from YE (p = 0.2039 and p = 0.1469, respectively).

However, closer examination indicates that urease activity alone cannot fully explain the observed mechanical performance. For instance, although the complete SP substitution group exhibited relatively high urease activity (Fig 3a), it yielded a substantially lower UCS (1.23 MPa) compared to the partial YE + SP group (2.49 MPa). This apparent discrepancy suggests that while urease activity is a prerequisite for initiating carbonate precipitation, the final mechanical properties are also profoundly influenced by the precipitation pathway and the quality of the precipitated carbonate, including its crystal morphology, spatial distribution, and the resulting cementation microstructure. Therefore, the YE + IYE formulation is better interpreted as achieving a more favorable balance between biochemical activity and cementation quality, rather than simply achieving a higher enzymatic level. Given that this formulation required only 34.47% of the control nitrogen-source cost, it demonstrates clear economic advantages while maintaining mechanical performance comparable to the control.

### 3.3. CaCO_3_ content analysis

**Fig 5:**
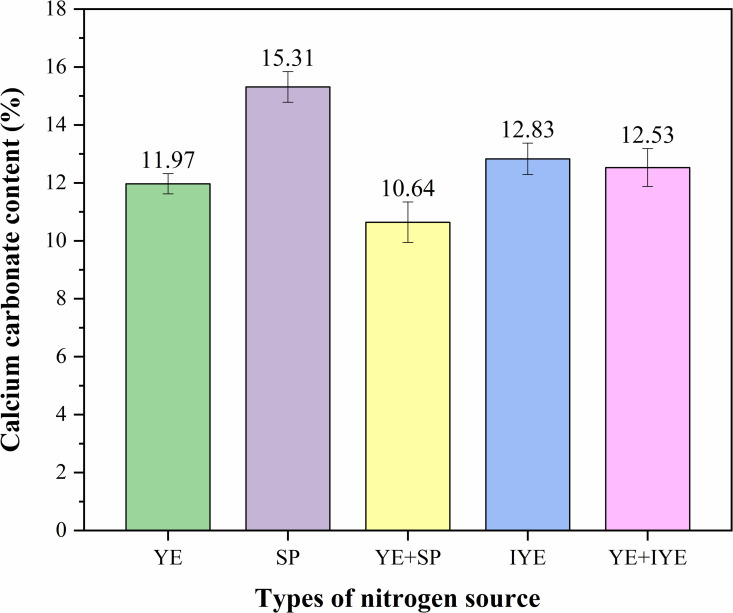
CaCO_3_ content of bio-cemented sand columns under different nitrogen source substitutions (mean ± SD, n = 3). YE (Control): pure YE (20 g/L); SP: SP (40 g/L, complete substitution); YE + SP: pure YE (5 g/L) + SP (15 g/L, partial substitution); IYE: IYE (20 g/L, complete substitution); YE + IYE: pure YE (5 g/L) + IYE (10 g/L, partial substitution).

Given that urease activity and UCS are not perfectly positively correlated, this section analyzes CaCO_3_ content to elucidate the factors influencing mechanical performance. As shown in Fig 5, most substitution groups exhibited higher CaCO_3_ content than the control (11.97%). The complete SP substitution group yielded the highest content (15.31%), while the partial SP substitution group (YE + SP) resulted in the lowest (10.64%); the IYE and YE + IYE groups showed moderate CaCO_3_ contents (12.53–12.83%).

From a mechanical performance perspective, a general trend suggests that higher CaCO_3_ contributes to greater UCS (e.g., YE+IYE’s 12.53% content was paired with the highest strength of 4.27 MPa). However, notable exceptions were observed: the YE + SP group showed moderate strength despite its low CaCO_3_ content, while the complete SP group exhibited low UCS comparable to that of other complete substitution groups, despite possessing the highest CaCO_3_ content. These observations suggest that UCS is not determined solely by the amount of precipitated CaCO_3_. Instead, the nitrogen source substitution strategy may influence the CaCO_3_ precipitation process and the resulting cementation effect, possibly through changes in crystal morphology, pore distribution, and other microstructural features. In addition, variations in urease activity under different substitution ratios (Fig 3) may affect the precipitation kinetics and crystal structure of CaCO_3_, ultimately impacting macroscopic strength. Therefore, CaCO_3_ polymorph distribution, spatial localization within pores, and microstructural characteristics may also contribute to strength development. The following sections (XRD, FTIR, and SEM) are used to test these hypotheses by examining whether nitrogen source substitution is associated with differences in these microstructural properties.

### 3.4. Nitrogen source-regulated CaCO_3_ precipitation and strength formation mechanism

To verify this hypothesis and elucidate how these compositional differences influence the properties of the precipitated carbonate, we subsequently analyzed the phase composition and microstructure of the precipitates.

#### 3.4.1. Crystal phase analysis.

**Fig 6:**
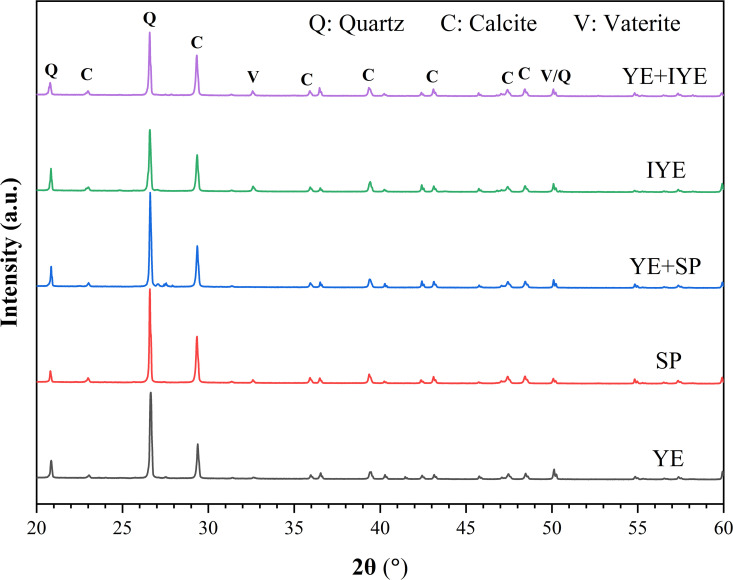
XRD patterns of bio-cemented sand under different nitrogen source substitutions.

XRD confirmed the presence of quartz (SiO_2_) and calcite (CaCO_3_) as the dominant crystalline phases in all samples (Fig 6). Characteristic diffraction peaks of calcite were observed at 29.4°, 36.0°, 39.4°, 43.1°, 47.5°, and 48.5° (2θ). Vaterite, identified by peaks at 27.0°, 32.8°, and 50.0° (2θ), was detected in the control and both IYE substitution groups (complete and partial). The presence of metastable vaterite in several groups warranted further evaluation of its potential influence on mechanical strength.

**Fig 7:**
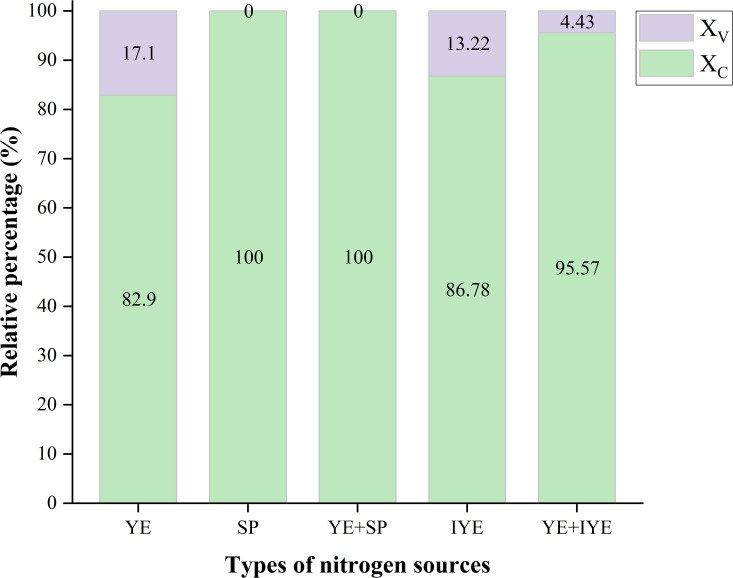
Polymorphic composition of CaCO_3_ precipitates under different nitrogen source substitutions. X_V_: molar ratio of vaterite; X_C_: molar ratio of calcite.

The results of the quantitative phase composition analysis are summarized in Fig 7. The YE control group contained 82.9% calcite (X_C_) and 17.1% vaterite (X_V_). Both the SP and YE + SP groups exhibited 100% calcite, indicating complete conversion to the stable polymorph. The IYE and YE + IYE groups showed calcite contents of 86.78% and 95.57%, with corresponding vaterite contents of 13.22% and 4.43%, respectively. The YE + IYE group exhibited both a high calcite proportion and the highest UCS. However, this does not mean that calcite content alone determines strength, because the SP-substituted groups also showed 100% calcite but much lower UCS. These results suggest that the mechanical performance of MICP-treated sand is controlled not only by the final polymorph composition, but also by how the crystals form and where they are deposited within the pore network. In the SP group, the calcite precipitates may have formed as relatively loose aggregates or in less effective locations for interparticle bridging, whereas in the YE + IYE group, the precipitation pathway appears to have favored the development of a denser and more continuous cementation network. Calcite contributes to mechanical strength by increasing material density through pore filling (reducing apparent porosity) and providing nucleation sites that facilitate further mineralization [39].

Notably, although the SP substitution groups (SP and YE + SP) achieved 100% calcite crystallization, their mechanical performance did not fully align with this, suggesting that other factors, such as crystal morphology, the spatial distribution of precipitates, and pore-filling efficiency, may also contribute to strength development.

#### 3.4.2. Functional group analysis.

**Fig 8:**
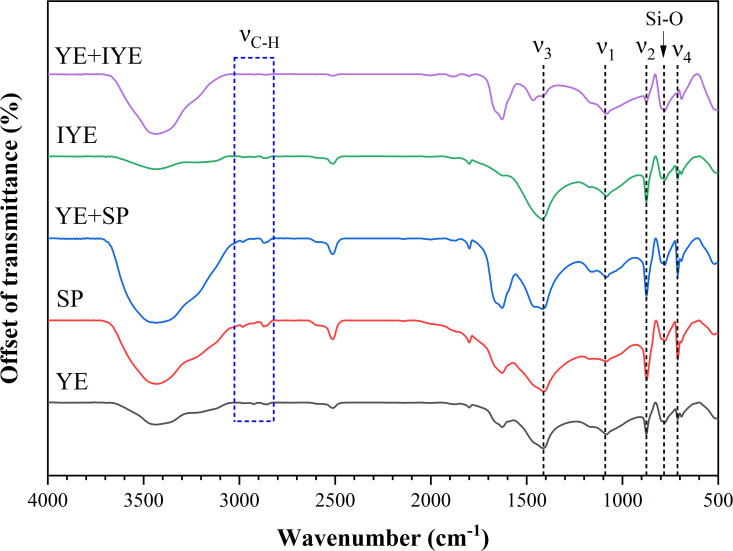
FTIR spectra of mineralized deposits under different nitrogen source substitutions. Spectra are offset for clarity.

To obtain complementary molecular-level insights, the chemical bonding environments were further analyzed by Fourier transform infrared (FTIR) spectroscopy. The FTIR spectra (Fig 8) corroborated the XRD results. All samples exhibited characteristic carbonate bands including asymmetric C-O stretching (ν_3_) at approximately 1412 cm^-1^, out-of-plane bending (ν_2_) at 875 cm^-1^, and in-plane bending (ν_4_) at 713 cm^-1^. Samples containing vaterite, as identified by XRD, showed noticeable broadening of the ν_3_ band around 1412 cm^-1^, contrasting with the sharper profile characteristic of pure calcite samples [33]. Peaks at 783 and 1087 cm^-1^ were attributed to Si-O vibrations from quartz. The broad band around 1640 cm^-1^ is associated with adsorbed water (H-O-H bending) in the KBr pellets. The broadening of the ν₃ band provides a spectroscopic indicator of vaterite co-existing with calcite.

In the high-wavenumber region (3600–3200 cm^-1^), all groups showed a broad absorption band, suggesting the presence of significant O–H or N–H stretching vibrations. The weak peaks at ~2853 and 2926 cm^-1^ are assigned to the C–H stretching vibrations of organic compounds, specifically the symmetric and asymmetric modes, respectively. In the mid-wavenumber range (2000–1500 cm^-1^), a series of peaks associated with carbonate vibrations (combination bands or overtones) was observed. In the low-wavenumber region (1000–500 cm^-1^), the distinct peak corresponding to the ν_4_ vibrational mode of calcite further confirmed its presence in all samples. The YE + IYE group also exhibited sharper and more defined peaks in this region, suggesting a higher calcite purity and improved crystallinity. Notably, the unique spectral features observed in the YE + IYE spectrum may be attributed to the interactions with organic templates derived from IYE, which may promote calcite formation [40]. In summary, the FTIR results support the XRD data and provide further evidence that nitrogen source substitution strategies considerably affect the molecular bonding environment and crystallinity of CaCO_3_ in bio-cemented sand.

#### 3.4.3. Microstructural analysis.

**Fig 9:**
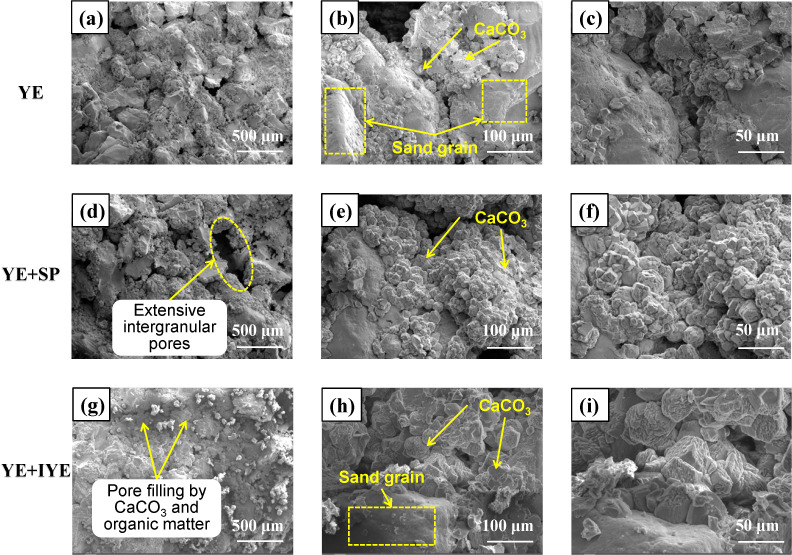
SEM micrographs of bio-cemented sand under different nitrogen source substitution strategies. Panels **(a–c)**, **(d–f)**, and (g–i) correspond to the control (YE), YE + SP, and YE + IYE groups, respectively. From left to right, the columns correspond to magnifications of 200 × , 1000 × , and 2000 × , respectively. YE is the control group using pure YE (20 g/L); YE + SP is the partial substitution group using pure YE (5 g/L) + SP (15 g/L); YE + IYE is the partial substitution group using pure YE (5 g/L) + IYE (10 g/L).

SEM analysis was conducted on the control and the representative partial substitution groups, with micrographs captured at 200 × , 1000 × , and 2000 × magnifications (Fig 9). Observations revealed that CaCO_3_ precipitated predominantly on sand grain surfaces and within intergranular pores, serving as the primary cementing agent. At lower magnifications (200× and 1000×), both the control and the IYE partial substitution group showed extensive pore infilling, with fine-scale cementation products occupying the remaining voids, thereby effectively densifying the cemented granular matrix [41].

In contrast, the SP partial substitution group exhibited higher visible porosity, which correlated with its lower UCS—samples with higher porosity consistently displayed lower mechanical strength. Higher magnification (2000×) revealed further microstructural distinctions: the IYE partial substitution specimen exhibited significantly fewer micron-scale pores and displayed densely intergrown, interlocking crystal formations. These results confirm that the IYE-based partial substitution strategy promotes enhanced microstructural densification, which in turn underpins its improved mechanical performance.

Taken together with the XRD results, the SEM observations indicate that polymorph composition should not be interpreted independently of microstructure. The small amount of vaterite detected in the YE + IYE group does not necessarily weaken the material; instead, it may reflect a different crystallization pathway that is more favorable for precipitation at particle surfaces and contact points, thereby forming a compact and effective load-bearing cementation framework. By contrast, even precipitates composed entirely of calcite cannot provide high strength if their spatial distribution and bonding architecture are unfavorable.

**Fig 10:**
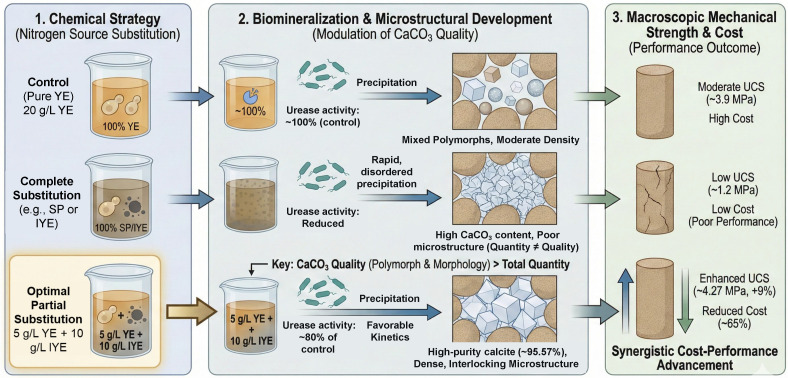
Schematic illustration of the nitrogen source substitution strategies and their material performance outcomes.

Based on the above XRD and SEM analyses, the critical role of nutrient formulation in determining the performance of bio-cemented sand is illustrated in Fig 10, which links the chemical strategy to microstructural development and macroscopic mechanical strength. While complete replacement of yeast extract (YE) with industrial alternatives (SP or IYE) was investigated, partial substitution with IYE (5 g/L YE + 10 g/L IYE) was identified as the optimal strategy. This strategy sustained robust bacterial metabolism (~80% of the control’s urease activity), thereby favoring calcite precipitation and promoting the formation of a dense, interlocking CaCO_3_ microstructure. The resulting effective cementation enhanced the UCS to ~4.27 MPa, a 9% improvement over the control, while simultaneously reducing nitrogen-source costs by ~65%.

### 3.5. Characterization of nitrogen-source feedstocks

**Fig 11:**
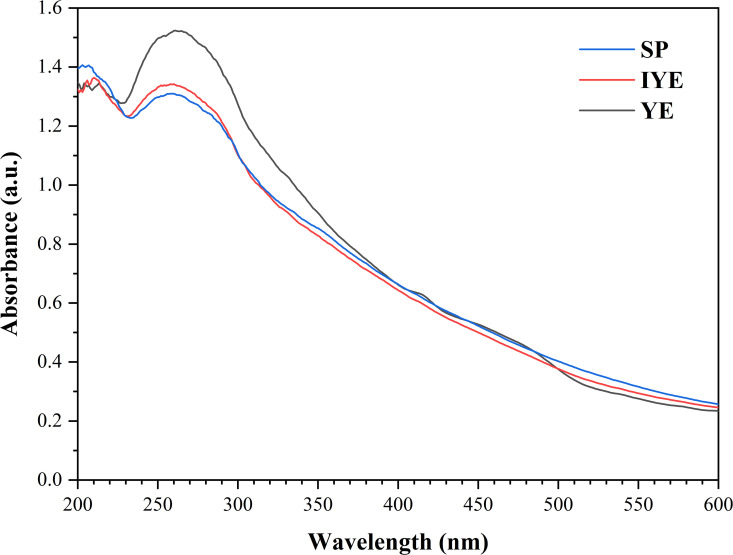
UV-Vis absorption spectra of YE, IYE, and SP.

**Fig 12:**
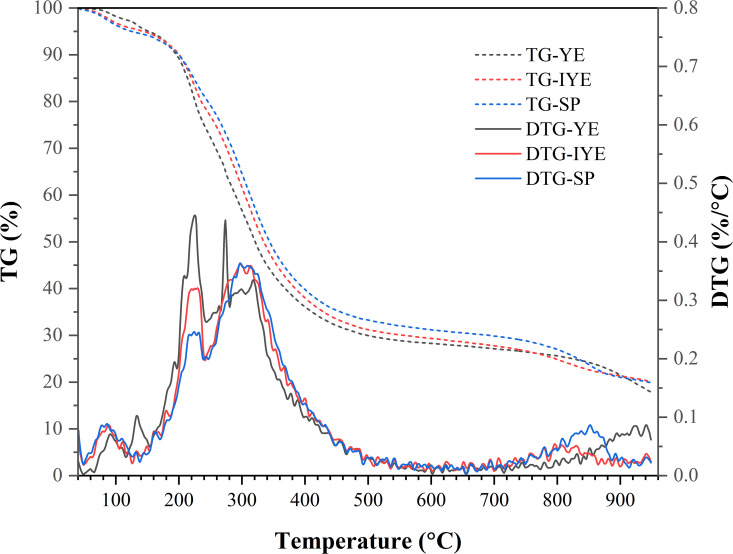
Thermal decomposition profiles of YE, IYE, and SP.

To elucidate the underlying causes of these divergent outcomes, we characterized the inherent physicochemical properties of the YE, IYE, and SP feedstocks themselves. Elemental (CHNOS) analysis (Table 3), UV-Vis spectroscopy (Fig 11), and TG-DTG analysis (Fig 12) collectively reveal systematic compositional and physicochemical differences among YE, IYE, and SP, which correlate with their distinct behaviors in MICP.

**Table 3:** Elemental composition (CHNOS) and mass ratios of YE, IYE, and SP (mean values of three independent measurements, n = 3).

Sample	C (wt%)	H (wt%)	N (wt%)	O (wt%)	S (wt%)	N/C Ratio (%)
**YE**	40.05	6.66	11.43	38.25	0.25	28.55
**IYE**	38.66	5.96	11.76	31.10	0.17	30.40
**SP**	37.61	5.84	12.25	31.69	0.20	32.58

YE exhibits the highest O content (suggesting a higher degree of oxygenation and potentially greater polarity/hydrophilicity), while SP contains the highest N content and N/C ratio; IYE shows intermediate values. Consistently, the UV-Vis spectra (200–600 nm) show a more pronounced absorption shoulder at 250–290 nm for YE, followed by IYE, with SP showing the lowest intensity, indicating a higher abundance of water-soluble UV-active organic constituents (e.g., aromatic/heteroaromatic chromophores). In contrast, SP and IYE display a slightly higher absorption tail above 500 nm, implying a more significant contribution from colored or more complex organic background components.

The TG–DTG profiles exhibit an initial mass loss around 80–120 °C, attributable to moisture release. Within the main decomposition range (150–350 °C), YE decomposes earlier and more sharply, with DTG features near ~225 °C and ~275 °C, indicating a larger fraction of mid-temperature thermally labile components. In contrast, IYE and SP display main DTG peaks at higher temperatures (~300 °C), suggesting more thermally stable and/or char-forming fractions. YE also leaves a slightly lower high-temperature residue (~950 °C) than IYE and SP, implying a comparatively lower background of inert (non-volatile) high-temperature residues.

The higher oxygenation, stronger UV absorption in the 250–290 nm region, and lower residue of YE suggest a greater fraction of readily soluble organic components in aqueous media, which can promote microbial metabolism and facilitate more homogeneous CaCO_3_ precipitation. Importantly, however, these feedstock differences do not imply that IYE or SP are intrinsically detrimental. Instead, they help explain the performance contrast between complete and partial substitution: complete replacement with SP/IYE may reduce the supply of rapidly bioavailable nutrients, whereas partial substitution (especially YE + IYE) leverages complementarity—a low dose of pure YE provides readily assimilable components to support urease-related metabolic activity, while industrial-grade sources supply cost-effective bulk nutrition and additional organics that may participate in adsorption/templating during mineralization. Consistent with this interpretation, the YE + IYE group achieved both high retained urease activity and the best cementation performance, indicating that the dosage and blending strategy, rather than the industrial source per se, govern the final cementation quality.

## 4. Conclusions

This study addresses a major economic constraint on the large-scale application of MICP—the high cost of nitrogen sources, which account for over 70% of cultivation expenses—by introducing a novel partial substitution strategy employing industrial-grade alternatives. Through systematic replacement of pure YE with standardized IYE and SP under defined substitution ratios, we successfully reconciled cost reduction with performance enhancement. The principal findings are summarized as follows:

(1) Balanced urease activity through metabolic-nutritional optimization.

The partial replacement design balances metabolic stability, maintained by a small amount of pure YE, with cost-efficient nutrition supplied by industrial-grade sources. This approach optimized urease activity, retaining 80% of the control activity while promoting favorable calcite precipitation kinetics, thus laying a foundation for high-quality CaCO_3_ formation.

(2) Superior mechanical performance governed by carbonate quality over quantity.

A key finding is that total CaCO_3_ content was decoupled from mechanical strength: the complete substitution group with SP yielded a high CaCO_3_ content (15.31%) but a low UCS (1.23 MPa); in contrast, the IYE-mediated partial substitution group achieved a much higher UCS (4.27 MPa) with a moderate CaCO_3_ content (12.53%). The superior strength is attributed to a favorable precipitation process that produced an effective cementation structure, characterized by a high calcite proportion together with more favorable crystal morphology, spatial distribution, and pore-filling behavior. These results indicate that final strength is governed by the coupled effects of polymorph composition and microstructural organization, rather than by CaCO_3_ content or polymorph type alone.

(3) Microstructural densification as the direct determinant of strength.

Microstructural analysis revealed that IYE substitution facilitates the development of dense calcite crystallization and an interlocking microstructure. As corroborated by SEM observations, this densification reduces pore space and improves particle bonding efficiency, providing a direct microstructural explanation for the superior mechanical behavior associated with high-quality CaCO_3_ formation.

(4) Synergistic cost-performance advancement.

The optimal nitrogen formulation (5 g/L pure YE + 10 g/L IYE) functions via a well-defined mechanism involving urease activity regulation, modulation of CaCO_3_ quality, and microstructural densification. This approach reduced costs by 65.53% while yielding a UCS of 4.27 MPa, which was statistically comparable to that of the pure YE control group. These results demonstrate a favorable synergy between cost efficiency and mechanical performance, highlighting the YE + IYE partial substitution strategy as the best-performing overall formulation in this study.

This study provides laboratory-scale validation of industrial nitrogen source substitution for MICP-treated sand columns, but several limitations should be noted. First, the results are based on small-scale indoor sand column tests and cannot fully represent field-scale reinforcement behavior. Second, cementation uniformity under practical conditions may also be affected by injection distance, soil heterogeneity, and local clogging. Third, the present work focused on short-term mechanical performance, and the long-term strength evolution and durability were not systematically evaluated. In addition, the effects of complex environmental factors, such as salinity, groundwater chemistry, temperature–humidity variation, and coupled field conditions, require further investigation before engineering application.

Overall, partial substitution with IYE represents a promising and cost-effective strategy for improving MICP performance, offering enhanced mechanical properties together with reduced material cost through better control of the biomineralization pathway. These findings provide a useful experimental basis for advancing industrial nitrogen source substitution in MICP, although further validation under larger-scale, long-term, and more complex environmental conditions is still needed before full engineering implementation.

## Supporting information

S1 File Stress-displacement curves. (DOC)
