# Supplementary material for: Industrial-grade nitrogen sources modulate CaCO3 polymorphs and strength in MICP-cemented sand: A structure–property study
Source: PLoS One. 2026 May 5;21(5):e0348780. doi: 10.1371/journal.pone.0348780 (PMC13143073; doi:10.1371/journal.pone.0348780)
Supplement: S1 File — (DOC) [file pone.0348780.s001.doc]

**Supporting Information for**

**Industrial-Grade Nitrogen Sources Modulate CaCO_3_ Polymorphs and Strength in MICP-Cemented Sand: A Structure–Property Study**

*The stress–displacement curves of the three parallel specimens in each group are provided in Fig. R1(a–e).*

(a) YE

(b) SP

(c) YE+SP

(d) IYE

(e) YE+IYE

**Fig. R1(a–e).** Stress–displacement curves of the three parallel specimens in each treatment group.
